# Modeling the movement of *Oecophylla smaragdina* on short-length scales in an unfamiliar environment

**DOI:** 10.1186/s40462-023-00426-w

**Published:** 2023-10-16

**Authors:** L. Charoonratana, T. Thiwatwaranikul, P. Paisanpan, S. Suksombat, M. F. Smith

**Affiliations:** 1 School of Physics, Suranaree University of Technology, Nakhon Ratchasima, 30000 Thailand; 2 NANOTEC-SUT Center of Excellence on Advanced Functional Nanomaterials, Suranaree University of Technology, Nakhon Ratchasima, 30000 Thailand; 3 School of Sport Science, Suranaree University of Technology, Nakhon Ratchasima, 30000 Thailand

**Keywords:** Animal movement, Weaver Ants, Thigmotaxis, Langevin Theory

## Abstract

The movement of individual weaver ants, of *Oecophylla smaragdina*, was previously tracked within an unfamiliar arena. We develop an empirical model, based on Brownian motion with a linear drag and constant driving force, to explain the observed distribution of ants over position and velocity. Parameters are fixed according to the isotropic, homogeneous distribution observed near the middle of the arena. Then, with no adjustable parameters, the model accounts for all features of the measured population distribution. The tendency of ants to remain near arena edges is largely explained as a statistical property of bounded stochastic motion though evidence for active wall-following behavior appears in individual ant trajectories. Members of this ant species are capable of impressive feats of collective action and long-range navigation. But we argue that they use a simplistic algorithm, captured semi-quantitatively by the model provided, to navigate within the confined region.

## Introduction

The social coherence of ant colonies has fascinated naturalists for centuries [1, 2]. Members of a colony work together to complete elaborate tasks. It requires the combined effort of many weaver ants, of *Oecophylla smaragdina* [3, 4] from tropical Asia and Australia, to bend and glue tree leaves while building their nest. This species, along with one other in the genus, is also notable for its colonies use of ‘living bridges’, made of hundreds of individuals, to negotiate gaps. Such collective action is only possible when individuals move in a precise manner and communicate, on both short and long length scales [5–7], to influence the motion of others. The weaver ants are known to employ a sophisticated system of communication, combining chemical signals with body motion to deliver distinct directives [3, 4, 8–10].

There is a large volume of literature on how the motion of one ant is influenced by messages received from another [2, 8, 11] and on the tools that an individual ant uses to navigate [12–23]. Desert ants [24, 25] in an unfamiliar environment use their view of the panorama [26] and path integration of their vector displacement [27] to navigate back to the nest. When foraging, they may carry out systematic searches of the local territory, moving in widening circles like other insects [28, 29], or merge navigational tactics with searching algorithms when they have incomplete knowledge of their whereabouts [30–32].

The movement of insects that do not have a certain destination is often modeled as a generalised random walk [33–36] similar to Brownian motion. Observed by Brown in 1827 and explained theoretically by Einstein and Langevin in the early 1900s [37–41], Brownian motion is exemplified by a small grain immersed in water that moves erratically because it receives impulses from collisions with neighboring molecules. Its velocity changes, like those of a crawling ant, are difficult to predict so they are treated as random quantities. While having this stochastic component, ant movement is also partly deterministic because the ant responds predictably to features in the local environment [42].

Ants are among many animals, including other insects [43], fish [44], rodents [45] and even humans during an evacuation [46] that exhibit wall-following. This is the tendency to move along a one-dimensional inhomogeneity within a two-dimensional space [47–49] (when the animal maintains bodily contact with the wall, it is termed thigmotaxis). The behavior has plausible adaptive value since a physical wall offers partial shelter and any 1D feature can be used as a directional guide [48–50]. Faced with a choice between two otherwise identical bridges, black garden ants select the one with a wall along its edge [51]. Rock ants tends to move parallel to distant walls [52] while desert ants follow the mid-lines between rows of shrubs [53]. Models of the Brownian motion of cockroaches [54] and harvester ants [55] in a confined space include position-dependent terms that represent an affinity for arena boundaries [56].

We previously carried out an experimental study [57] of the motion of a single weaver ant confined to a featureless, unfamiliar arena. An individual ant was snatched from a nest, placed on a square ceramic tile bounded by water, and its motion tracked. The only macroscopic inhomogeneity of the arena is its boundary, to which the ant tended to remain close. We chose the weaver ant because of its propensity for correlated action and its availability in our area.

In this paper we develop a model, based on Langevin theory, of the movement algorithm exhibited by the weaver ants and compare the results of numerical simulations and analytical calculations to experiment. Random velocity changes of model ants are governed by a fixed probability distribution augmented by deterministic drag and driving forces along the direction of motion. After it encounters the arena boundary and is forced to stop, the model ant immediately resumes its fixed movement algorithm. The model reproduces qualitative features of the observed distribution of ants over position and velocity without adjustable parameters.

We use this model to investigate whether an enhanced ant density at arena edges is evidence that ants follow walls actively, by modifying their movement algorithm when they are close to the edge and/or preferentially moving towards this edge, or whether it can be explained as a result of passive stochastic motion bounded by the arena. Our model includes no active wall-following behavior: model ants use the same movement algorithm near edges as in the open arena and are no more likely to turn towards a boundary as away from it. They nonetheless exhibit a high probability to be found within a short distance from the arena edge, in agreement with experiment. Going beyond this description of the population distribution, we consider below the residence times of individuals in the near-edge region, for both real and model ants, to isolate active wall-following behavior and evaluate its significance.

The paper is organized in the following way. In section “Experimental Results” we summarize the results of our previous experiment and discuss a new analysis of the ant distribution data. In section “Model of ant motion” we develop a model based on the distribution of ants in the arena interior, away from the boundaries. In section “Analytic approximation” we study the model analytically in order to better understand its key properties. In section “Results and discussion” we present the results of numerical simulations of the model and compare them to the experimental data in detail. We discuss the implications of the work in section “Results and discussion” before concluding in section “Conclusions”.

## Experimental results

Previously we described an experiment (see Ref. [57] for details) in which individual weaver ants, from *Oecophylla smaragdina*, were captured from one of many nests located on campus at Suranaree University of Technology, Thailand. All ants were the aggressive [58] female worker ants that comprise the majority of the colony. Ants were transported to the laboratory and one was released into an arena, a dry ceramic tile surrounded by water, where its motion was tracked via a stationary camera for five minutes. The time between an individual being captured and its observation completed was between ten and forty minutes, after which it was returned to its nest. The ant is unlikely to be motivated by hunger during the trial, but we do not speculate about its purpose for moving about the arena. Its motion was nearly continuous, without apparent change in its nature, throughout the trial.

A single ant was tracked as it moved on a square tile of length $L=30$ cm centered on the origin and surrounded by a channel of water that it never attempted to cross. The tile and camera were located at a fixed position within a small room. The arena is defined by $|x|<L/2$ and $|y|<L/2$ where the orientation of the *x* and *y* axes was parallel to the edges of the tile. The ant position ${{\textbf {x}}}(t)=(x[t],y[t])$ was measured at time intervals $\Delta t=1/15$ s over a duration $T=4500\Delta t=300$s, and the velocity ${{\textbf {v}}}(t)=({{\textbf {x}}}[t+\Delta t]-{{\textbf {x}}}[t])/\Delta t$ and change in velocity $\Delta {{\textbf {v}}}(t)={{\textbf {v}}}(t+\Delta t)-{{\textbf {v}}}(t)$ were determined. The experiment was repeated on $N=60$ individual ants, using many similar tiles, each washed and re-used multiple times.

**Fig. 1:**
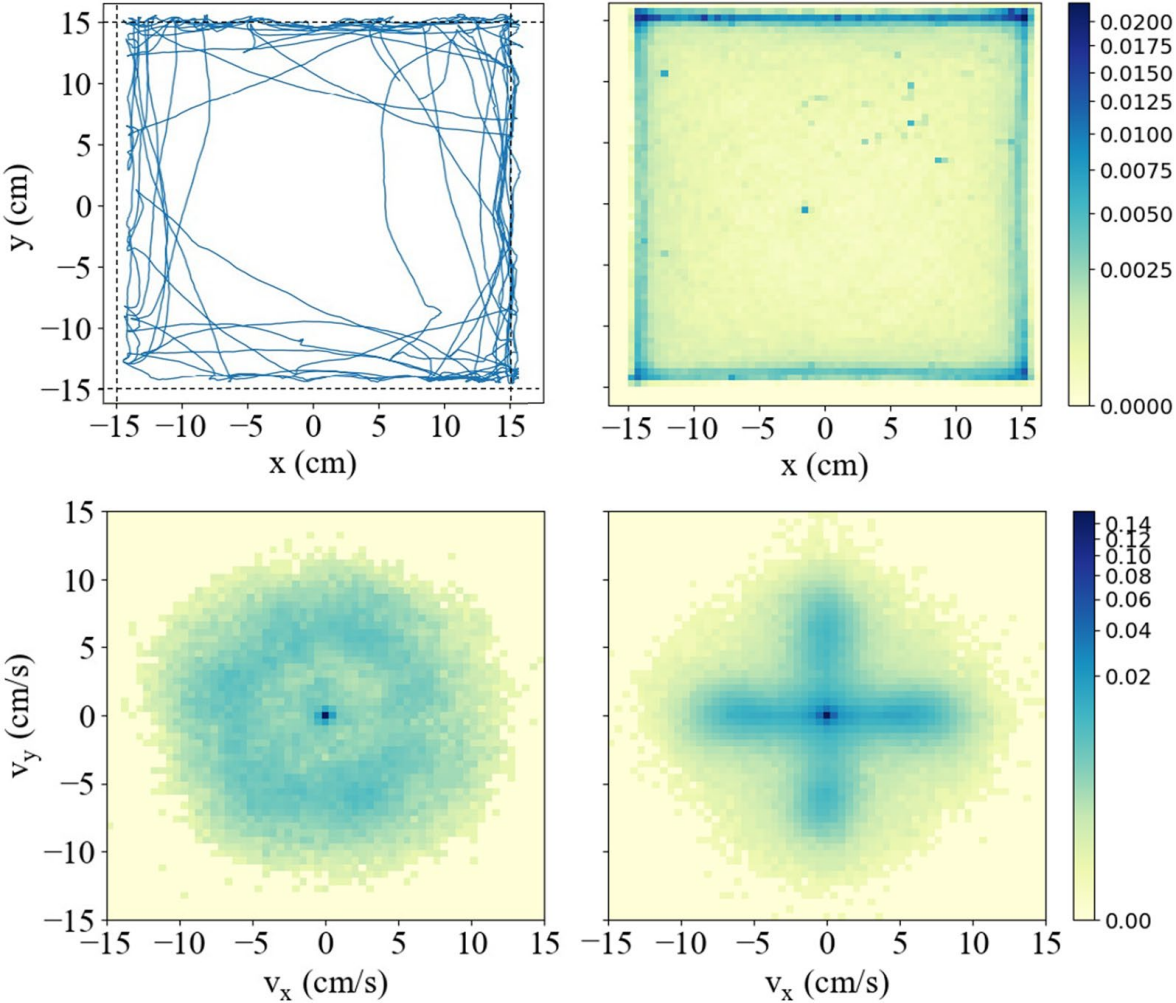
In the top left panel we show the trajectory of a single ant during a typical trial, with all points ${{\textbf { x}}}(t)=(x[t],y[t])$ measured during a trial of duration $T=300$ s connected with the blue curve. The other panels display ‘heat’ maps of the normalized distribution over position *n*(*x*, *y*) and velocity $P(v_x,v_y)$. The color scales indicate the value of *n*(*x*, *y*) in units of $\textrm{cm}^{-2}$ and $P(v_x,v_y)$ in units $(\mathrm {cm/s})^{-2}$. Upper right: *n*(*x*, *y*) is largest near boundaries, decreases rapidly and then remains constant in the interior. Lower left: $P(v_x,v_y)$ for ants in the arena interior (further than 3 cm from a boundary) is isotropic with a non-monotonic speed dependence. Lower right: $P(v_x,v_y)$ for ants within 3 cm of the boundary looks like a ‘plus’ sign because ants move along arena edges

Sample data, showing the trajectory of ${{\textbf {x}}}(t)$ for one ant over the full duration *T*, is shown in the top left panel of Fig. 1. The ant was released into the arena by inverting a plastic container near the middle of the arena, waiting for the ant to crawl out, then removing the container and starting the camera. For the trial shown, the first position measurement was ${{\textbf {x}}}(0)\approx (8,8)$ cm and the ant mainly stays close to the arena boundary, which was typical.

All data were combined into a single set, treated as a statistical distribution. By studying single ant motion using this distribution, we are averaging over the peculiarities of individuals. The *j*th ant has a position ${{\textbf {x}}}_j(t)$ and corresponding velocity ${{\textbf {v}}}_j(t)$, where $t=0,\Delta t,2\Delta t,..T$. We have an experimental probability distribution defined by

1 $$\begin{aligned} \Pi ({{\textbf {x}}},{{\textbf {v}}},t)=\frac{1}{N}\sum _{j=1}^N\delta ({{\textbf { x}}}_j-{{\textbf {x}}})\delta ({{\textbf {v}}}_j-{{\textbf {v}}}). \end{aligned}$$

The time dependence of $\Pi ({{\textbf {x}}},{{\textbf {v}}},t)$ was weak for all $t>>\Delta t$, so the time-averaged quantity $\Pi ({{\textbf {x}}},{{\textbf {v}}})=T^{-1}\int dt \Pi ({{\textbf {x}}},{{\textbf {v}}},t)$ can be interpreted as an equilibrium value. Equation 1 is equivalent to a Boltzmann distribution function [59] in many-particle physics, but is here used to describe the motion of many individual ants moving in the arena at different times (i.e. different trials). The model below cannot be applied to a population of ants in the arena together because it includes no ant-ant interactions.

The density $n({{\textbf {x}}})$ and velocity distribution $P({{\textbf {v}}})$ are defined by

2 $$\begin{aligned} n({{\textbf {x}}})=\int d{{\textbf {v}}} \Pi ({{\textbf {x}}},{{\textbf {v}}}),\;\;\;P({{\textbf {v}}})=\int d{{\textbf {x}}} \Pi ({{\textbf {x}}},{{\textbf {v}}}). \end{aligned}$$

The limits of the velocity integrals are $\pm \infty$, those of the position integrals are $\pm L/2$, and $\Pi ({{\textbf {x}}},{{\textbf {v}}})$, $n({{\textbf {x}}})$, and $P({{\textbf {v}}})$ are all normalized probability distributions.

A two-dimensional map of $n({{\textbf {x}}})$ is shown at the top right of Fig. 1. The density decreases rapidly with distance from the boundary, approaching a value $n({{\textbf {x}}})\approx n_{\textrm{int}}$ that is constant throughout the interior. Ants spent only 20 percent of their time at positions further than 3 cm from the boundary, with $n_{\textrm{int}}\approx 4\cdot 10^{-4}$cm^-2^. They spent most of their time near the boundaries: within 3 cm of $x=\pm L/2$ and $y=-L/2$ the average density was $5.3 n_{\textrm{int}}$ while within 3 cm of $y=L/2$ it was $8.2 n_{\textrm{int}}$. The preferred $y=L/2$ boundary was positioned closest to the window in the laboratory room.

The map of the velocity distribution $P({{\textbf {v}}})$ is shown at the bottom of Fig. 1. The data has been broken up into two subsets: (i) ants in the interior and (ii) within 3 cm from a boundary. The former distribution is isotropic and depends only on speed $v=|{{\textbf {v}}}|$. *P*(*v*) initially decreases to a local minimum then increases, exhibiting a circular shoulder, before falling off at high speed. For ants near the boundary, $P({{\textbf {v}}})$ has four arms that correspond to members moving parallel to the square edges.

**Fig. 2:**
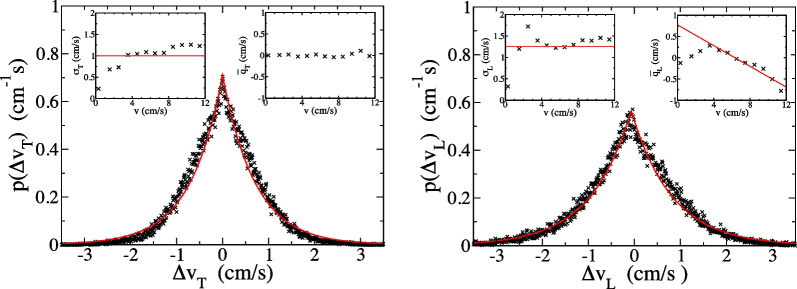
Fits of Eq. 4 to the experimental distribution of velocity changes $\Delta {{\textbf {v}}}$ for ants in the arena interior. Left: the distribution $p(\Delta v_T)$ for the component $\Delta v_T$ of $\Delta {{\textbf {v}}}$ that is perpendicular to velocity. The insets show the standard deviation *σ*_*T*_ and mean $\Delta v_T$ as a function of speed: the latter always zero. Right: $p(\Delta v_L)$ for the component $\Delta v_L$ of velocity change parallel to velocity. The insets show the standard deviation *σ*_*L*_ and mean of $\Delta v_L$ versus speed and their linear fits. Most data falls within the range of speed where this linear approximation is roughly valid

The plots in Fig. 1 provide an overview of ant behavior during the experiment. We develop a model of this behavior using the measured distribution of velocity changes within the arena interior. Data for the interior is the minority, but is simplest to analyze because motion is not affected by boundaries. So, we consider $\Delta {{\textbf {v}}}$ measured when ants were more than 3 cm from the boundary. In coordinates relative to velocity $\hat{{{\textbf {v}}}}$, it is given by

3 $$\begin{aligned} \Delta {{\textbf {v}}}=\Delta v_T\hat{{{\textbf {z}}}}\times \hat{{{\textbf { v}}}}+\Delta v_L\hat{{{\textbf {v}}}} \end{aligned}$$

where $\Delta v_T$ affects direction, with a positive value corresponding to a left turn, and $\Delta v_L$ changes speed (the vector $\hat{{\textbf {z}}}$ points up from the arena).

We use the same function *p*(*q*) to fit the distribution over $\Delta v_T=q$ or $\Delta v_L=q$, it is

4 $$\begin{aligned} p(q)=\frac{1}{\sqrt{2\sigma ^2}}\textrm{exp}\left( -\sqrt{\frac{2(q-\bar{q})^2}{\sigma ^2}}\right) , \end{aligned}$$

where the mean $\bar{q}$ and standard deviation $\sigma$ are the fitting parameters. The distribution over $\Delta {{\textbf {v}}}$ depends weakly on position within the arena interior. It does vary according to how fast the ant was moving when the velocity change occurred, so we sub-divided data for the interior according to speed *v*. The results are shown Fig. 2.

For velocity changes normal to motion $\Delta v_T$, shown on the left side of Fig. 2, Eq. 4 appears to provide a good fit to the distribution. In the insets we plot the best-fit values for $\sigma$ and $\bar{q}$ versus speed. The standard deviation has an average value of $0.996\pm 0.007$ cm/s, where the uncertainty is statistical. It can be approximated by a constant $\sigma =\sigma _T\approx 1.00$ cm/s for, while it deviates from this value at low speeds, the dominant fraction of the data falls within the region where $\sigma$ is speed-independent. The best-fit value for the mean $\bar{q}$ is always zero within error.

We checked whether $\Delta v_T(t)$ values for different times are independent by calculating the Pearson correlation coefficient [60] of $\Delta v_T(t)$ with $\Delta v_T(t+3\Delta t)$ and found a value of 0.01, indicating weak correlations. (We could not consider $\Delta v(t)$ values more closely-spaced in time because, according to their definition, $\Delta t\Delta v(t)\equiv x(t+2\Delta t)-2x(t+\Delta t)+x(t)$, they share *x*(*t*) data points.) So $\Delta v_T(t)$ for each time *t* can be regarded as an independent variable with a distribution *p*(*q*), a mean $\bar{q}=0$ and root-mean-square $\sigma _T$.

The distribution of changes parallel to velocity $\Delta v_L$ is shown on the right in Fig. 2. The fit with Eq. 4 appears reasonable, and the standard deviation $\sigma =\sigma _L\approx 1.25$cm/s can again be treated as a constant (its average value was $1.251\pm 0.008$ cm/s). But the mean $\bar{q}\ne 0$ has a characteristic *v* dependence, plotted in the upper-right inset. It increases with *v* to a small positive maximum before decreasing linearly into negative values. The majority of the data set falls within the range of speed where $\bar{q}$ is linearly decreasing and the linear fit shown is relevant. Below, we will assume that $\Delta v_L$ is composed of two terms, one random and the other deterministic. The latter results in the non-zero mean of $\Delta v_L$ and some positive correlation between velocity changes at nearby times.

## Model of ant motion

The distribution of velocity changes in the arena interior suggests that a simple model, based on Brownian motion, may be applicable. We assume that the observed motion of the ant in the interior is characteristic of its movement algorithm. The arena boundaries will be treated as constraints that do not otherwise modify movement. A model ant has a position and velocity that can be updated in simulations according to

5 $$\begin{aligned} {{\textbf {x}}}_j(t+\Delta t)={{\textbf {x}}}_j(t)+{\textbf {v}}_j(t)\Delta t,\;\;{\textbf {v}}_j(t+\Delta t)={\textbf {v}}_j(t)+{\textbf {F}}_j(t)\Delta t+{\textbf {q}}_j(t), \end{aligned}$$

where the second equation corresponds to Newton’s law for a particle of unit mass. The term ${\textbf {F}}_j(t)={\textbf {F}}({\textbf {v}}_j[t])$ is a deterministic force while ${\textbf {q}}_j(t)$ is a random impulse occurring each time step. Equation 5 is the basic rate equation of the Langevin theory of Brownian motion.

The force is written as

6 $$\begin{aligned} {\textbf {F}}({\textbf {v}})=-\frac{1}{\tau _D}{} {\textbf {v}}+\frac{v_0}{\tau _D}\hat{{\textbf {v}}} \end{aligned}$$

where the first term is a linear drag force, with a time constant $\tau _D$, and the second is a constant driving force in the forward direction, with a speed constant $v_0$. The impulse ${\textbf {q}}_j(t)={\textbf {q}}=q_T\hat{{\textbf {z}}}\times \hat{{\textbf {v}}}+q_L\hat{{\textbf {v}}}$ where $q_L$ is a random number governed by Eq. 4 with $\sigma =\sigma _L$ and $\bar{q}=0$ while $q_T$ is a random number governed by Eq. 4 with $\sigma =\sigma _T$ and $\bar{q}=0$. Since the mean impulse ${\textbf {q}}$ is zero, an average over all model ants with a given velocity ${\textbf {v}}_j(t)={\textbf {v}}$ results in

7 $$\begin{aligned} \langle \Delta {\textbf {v}}\rangle = \Delta t {\textbf {F}}({\textbf {v}}), \end{aligned}$$

where angular brackets denote this average. We can use the linear fit to the mean $\Delta {\textbf {v}}$, shown in the insets of Fig. 2, to obtain $\tau _D=0.55$ s and $v_0=6.3$ cm/s. The effects of the deterministic force are small during a single time step, since $|{\textbf {F}}|\Delta t<<\sigma _L,\sigma _T$, but are important on long time scales since they add constructively.

While we have based the movement algorithm on ant motion in the interior, we have to model the response of an ant to the boundary in order to compare simulations with data. We used the following protocol for the ant-boundary interaction. In simulations, if ${{\textbf { x}}}(t+\Delta t)$ is found outside the arena then we replace it with the nearest position on the boundary and set the velocity component normal to the boundary equal to zero. The position and velocity parallel to the boundary are not modified. With the subsequent time step, position and velocity are updated via Eq. 5 with the only difference being that the random velocity change cannot take the ant immediately back out of the arena: if the boundary is on the ant’s right side then $q_T(t+\Delta t)$ must be positive. This latter constraint, a detail that does not change any qualitative results, was used to prevent model ants from briefly sliding along the boundary, which seemed artificial.

The response of the model ant to the boundary is a key feature of our study, so we briefly consider a few alternative approaches. We could have analysed in detail the observed motion of the ant near the boundary and attempted to model it. This would require numerous empirical parameters and reduce the entire exercise to a fit of measured motion within our particular experimental configuration. We sought instead a minimal model that might have some predictive value. That said, while we updated the model ant velocity at the $x=L/2$ boundary according to $(v_x,v_y)\rightarrow (0,v_y)$, there are other simple procedures that could have been used. One would have model ants reflecting elastically off boundaries like billiard balls, $(v_x,v_y)\rightarrow (-v_x,v_y)$. This was dismissed because it cannot give rise to the measured distribution and seems to take the analogy with Newtonian particles to an absurd extreme. Another, to have ant speed vanish at the boundary $(v_x,v_y)\rightarrow (0,0)$, was rejected because it does not allow rapid motion along the boundary, which is seen at a first glance of the experiment. Any more sophisticated response of the ant to a boundary encounter, such as pausing for some time or changing direction in a particular way, would employ more fitting parameters.

Finally, one could introduce wall-following to the model by hand using a position-dependent deterministic force ${\textbf {F}}({\textbf {x}},{\textbf {v}})$ that attracts ants to the boundary at short distances. We avoided this for two reasons. First, such a force would introduce new fitting parameters that characterize its strength and range. Second, we wanted to investigate the possibility that the observed distribution of ants could be realized by a model that did not include any active wall-following behavior. Here we define *active* wall-following as a position-dependent algorithm that favors motion towards or along the boundary. In contrast, a model like ours that predicts an increased ant density at boundaries without employing a position-dependent movement algorithm could be said to exhibit *passive* wall-following behavior. Overall, we view the protocol we adopted as the simplest one that had any chance of reproducing the observed distributions. The model ant is forced to stop at the boundary but then resumes its motion, using the same algorithm it employs in the open arena, as if nothing happened.

The simulation was carried out many times and the distribution extracted from the numerical data and time-averaged. We usually started each simulated trial with the initial conditions ${\textbf {x}}_j(0)={\textbf {v}}_j(0)=0$ and the ant’s body orientation equally likely to be in any direction, but the equilibrium distribution was independent of initial conditions. The values of the model parameters: $\sigma _T$, $\sigma _L$, $\tau _D$, $v_0$ were all obtained experimentally from the distribution of $\Delta {\textbf {v}}$ in the arena interior. The model has no free parameters and the fixed parameters can only be properties of the movement algorithm for an ant in an unbounded arena. A comparison between the simulated and measured distributions in the bounded arena is thus meaningful, i.e. the model is falsifiable.

## Analytic approximation

### The rate equation

An approximate rate equation for the model detailed above is presented in this section. The goal is to obtain some analytic understanding of its properties. Particularly, we explain the length and speed scales of the model, which can be associated with those observed in the data. Using Eqs. 1, 5 and 6, we write the distribution at time $t+\Delta t$ as

8 $$\begin{aligned} \Pi ({\textbf {x}},{\textbf {v}},t+\Delta t)=\int d{\textbf {q}}p({\textbf {q}})\bigg (\frac{1}{N}\sum _j\delta ({\textbf {x}}_j+{\textbf {v}}_j\Delta t-{\textbf {x}})\delta ({\textbf {v}}_j+{\textbf {F}}_j\Delta t+{\textbf {q}}-{\textbf {v}})\bigg ). \end{aligned}$$

The probability that a given member receives an impulse ${\textbf {q}}$ within a time step is $d{\textbf {q}}p({\textbf {q}})$. We have

9 $$\begin{aligned} \int {{\textbf {dq}}} p({\textbf {q}})=1,\;\;\int {{\textbf {dq}}} p({\textbf {q}}){\textbf {q}}=0, \;\int {{\textbf {dq}}} p({\textbf {q}})q_L^2=\sigma _L^2,\;\;\int {{\textbf {dq}}} p({\textbf {q}})q_T^2=\sigma _T^2 \end{aligned}$$

where $q_L$ and $q_T$ are the components of ${\textbf {q}}$ that are parallel and perpendicular to ${\textbf {v}}$, respectively. The limits of the $q_L$ and $q_T$ integrals in Eq. 9 are $\pm \infty$.

If we drop terms of order $\Delta t^2$ and $\Delta t|{\textbf {q}}|$ then the first Dirac delta function in Eq. 8 can be replaced by $\delta ({\textbf {x}}_j+{\textbf {v}}\Delta t-{\textbf {x}})$. The second can be rewritten as

10 $$\begin{aligned} \delta ({\textbf {v}}_j+{\textbf {F}}_j({\textbf {v}}_j)\Delta t+{\textbf {q}}-{\textbf {v}})=\frac{\delta ({\textbf {v}}_j+\Delta t{\textbf {F}}({\textbf {v}})+{\textbf {q}}-{\textbf {v}})}{|1+\Delta t\frac{\partial }{\partial {\textbf {v}}}\cdot {\textbf {F}}({\textbf {v}})|}, \end{aligned}$$

where we used the property $\delta (f[x])=\delta (x-x_0)/|df/dx|_{x=x_0}$ with $f(x_0)=0$. Substituting these expressions and rearranging, we have

11 $$\begin{aligned} \Pi ({\textbf {x}},{\textbf {v}},t+\Delta t)=\left( 1-\Delta t\frac{\partial }{\partial {\textbf {v}}}\cdot {\textbf {F}}({\textbf {v}})\right) \int d{\textbf {q}}p({\textbf {q}})\Pi ({\textbf {x}}-{\textbf {v}}\Delta t,{\textbf {v}}-{\textbf {q}}-\Delta t{\textbf {F}}({\textbf {v}}),t). \end{aligned}$$

Now carrying out an expansion to first order in $\Delta t$ and second order in ${\textbf {q}}$ we get

12 $$\begin{aligned} \frac{\partial \Pi }{\partial t}=\frac{\partial }{\partial {\textbf {v}}}\cdot \bigg (-{\textbf {F}}\Pi \bigg )-{\textbf {v}}\cdot \frac{\partial }{\partial {\textbf {x}}}\Pi +\Delta \Pi _{\textrm{R}}/\Delta t. \end{aligned}$$

The last term, the average over squared random impulses, is $\Delta \Pi _{\textrm{R}}/\Delta t= \Lambda _{xx}+\Lambda _{yy}+\Lambda _{xy}$ with

13 $$\begin{aligned} \Lambda _{xx}= & {} \left( \frac{\sigma _L^2}{2\Delta t}\cos ^2\theta +\frac{\sigma _T^2}{2\Delta t}\sin ^2\theta \right) \frac{\partial ^2}{\partial v_x^2} \Pi ({\textbf {x}},{\textbf {v}},t) \end{aligned}$$

14 $$\begin{aligned} \Lambda _{yy}= & {} \left( \frac{\sigma _L^2}{2\Delta t}\sin ^2\theta +\frac{ \sigma _T^2}{2\Delta t}\cos ^2\theta \right) \frac{\partial ^2}{\partial v_y^2} \Pi ({\textbf {x}},{\textbf {v}},t) \end{aligned}$$

15 $$\begin{aligned} \Lambda _{xy}= & {} \left( \frac{\sigma _L^2}{2\Delta t}-\frac{\sigma _T^2}{2\Delta t}\right) \sin 2\theta \frac{\partial ^2}{\partial v_x\partial v_y} \Pi ({\textbf {x}},{\textbf {v}},t) \end{aligned}$$

where $${\textbf {v}}=(v_x,v_y)=v(\cos \theta ,\sin \theta )$$.

It is convenient to express this equation in dimensionless coordinates. We have parameters with the units of speed $v_\infty$ and distance $\ell$, and a dimensionless measure of anisotropy $\alpha$ that are

16 $$\begin{aligned} v_\infty ^2=\frac{\sigma _L^2\tau _D}{2\Delta t},\;\;\;\;\;\ell =v_\infty \tau _D,\;\;\;\;\; \alpha =\frac{\sigma _L^2-\sigma _T^2}{\sigma _L^2}. \end{aligned}$$

Dimensionless coordinates for velocity ${\textbf {u}}={\textbf {v}}/v_\infty$, position ${\textbf {r}}={\textbf {x}}/\ell$, time $\tau =t/\tau _D$, and force ${\textbf {f}}={\textbf {F}}\tau _D/v_\infty =-{\textbf {u}}+u_0{\hat{{\textbf {u}}}}$, with $u_0=v_0/v_\infty$, are introduced. The size of the arena is $R=L/\ell$. The dimensions of $\Pi ({\textbf {x}},{\textbf {v}},t)$ can be removed by multiplying it by $v_\infty ^2\ell ^2$. The dimensionless rate equation for the distribution $\Pi ({\textbf {r}},{\textbf {u}},\tau )$ is given by

17 $$\begin{aligned} \frac{\partial \Pi }{\partial \tau }=\frac{\partial }{\partial {\textbf {u}}}\cdot \left( -{\textbf {f}}\Pi +\frac{\partial }{\partial {\textbf {u}}}\Pi \right) -{\textbf {u}}\cdot \frac{\partial }{\partial {\textbf {r}}}\Pi -\alpha \left( \frac{1}{u^2}\frac{\partial ^2 \Pi }{\partial \theta ^2}+\frac{1}{u}\frac{\partial \Pi }{\partial u}\right) . \end{aligned}$$

The equilibrium distribution $\Pi ({\textbf {r}},{\textbf {u}})$ solves Eq. 17 with the left side equal to zero.

The model equilibrium distribution has two speed scales, $v_0$ and $v_\infty \approx 2.5$cm/s and one length scale $\ell \approx 1.4$cm. The value $v_0$, at which the driving and drag forces balance, is a mean speed for members moving in a given direction. The effect of random impulses are contained within $v_\infty$, the mean-square deviation of velocity from this mean. An ant remains approximately localized within a region of width equal $\ell$ because it is unlikely to travel this far without turning around. The anisotropy factor is $\alpha \approx 0.36$.

### Equilibrium distribution for a homogeneous, unbounded arena

We can study the model in the simple case of an unbounded arena with area $R^2$. (The actual, more difficult, case of a bounded arena is treated approximately in Appendix B). This is done by imposing periodic boundary conditions and can be imagined as the 2D arena stretched over a closed 3D shape. The equilibrium distribution $\Pi ({\textbf {r}},{\textbf {u}})$ is proportional to that for an arena of infinite size: every position and direction is symmetry-equivalent so the distribution, independent of ${\textbf {r}}$ and $\theta$, depends only on speed *u*.

For isotropic impulses, $\alpha =0$, the distribution satisfies

18 $$\begin{aligned} 0=\frac{\partial }{\partial {\textbf {u}}}\cdot \left( -{\textbf {f}}\Pi +\frac{\partial }{\partial {\textbf {u}}}\Pi \right) , \end{aligned}$$

which is solved to give $\Pi ({\textbf {r}},{\textbf {u}})=\Pi (u)\propto \textrm{e}^{-u^2/2+u_0u}$. (The second order differential equation has two independent solutions but we must choose the one that vanishes at large speed.) As a function of speed, in variables with units, this distribution has shoulder peaks at $v=\pm v_0$, each with a half-width $v_\infty$.

For anisotropic impulses, the equilibrium distribution is still independent of $\theta$. This is ensured by symmetry if the initial state of the ensemble is uniformly distributed over all ant-body orientations. A normalized solution to the rate equation for $\alpha \ne 0$ is

19 $$\begin{aligned} \Pi ({\textbf {r}},{\textbf {u}})=\Pi (u)=\frac{1}{2\pi N_0 R^2}\exp \left( \frac{-u^2+2u_0u+2\alpha \ln u}{2}\right) \end{aligned}$$

with the normalization constant

20 $$\begin{aligned} N_0=\int _0^\infty du u \exp \left( -u^2/2+u_0u+\alpha \ln u\right) . \end{aligned}$$

The anisotropy $\alpha$ of the random impulses, while not having a huge effect on the distribution, shifts the position of the shoulders to slightly higher speed. They are centered on $u=\pm v_s/v_\infty$ where

21 $$\begin{aligned} v_s=\frac{v_0+v_\infty \sqrt{(v_0/v_\infty )^2+4\alpha }}{2}. \end{aligned}$$

So the shoulder peak position $v_s\approx 6.7$cm/s

## Results and discussion

We compare the equilibrium distributions obtained from model simulations and experiment. First, consider the one-dimensional density and velocity distributions:

22 $$\begin{aligned} n(x)=\int _{-L/2}^{L/2} dy n(x,y)\;\;,\;\;P(v_x)=\int _{-\infty }^\infty dv_y P(v_x,v_y). \end{aligned}$$

and their counterparts *n*(*y*) and $P(v_y)$.

**Fig. 3:**
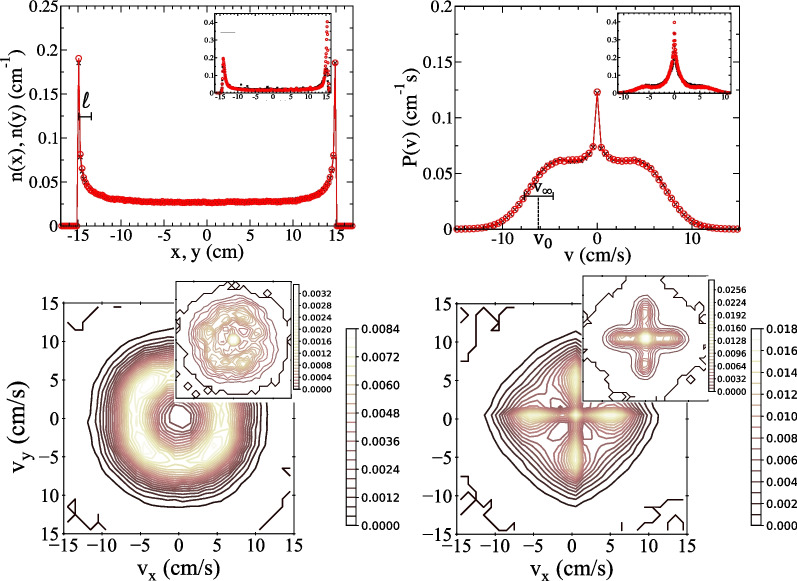
Comparing the simulated and measured distributions. The model parameters are $\tau _D = 0.55$ s, $v_0 = 6.3$ cm/s, $\sigma _L = 1.25$ cm/s and $\sigma _T = 1.00$ cm/s (the distance $\ell$, and speeds $v_s$ and width $v_\infty$ are given in Eqs. 16, 21). Top left: the density *n*(*x*) versus position *x* (black symbols) and *n*(*y*) versus position *y* (red) of ants in the simulation (main panel) and experiment (inset). Top right: the distribution over velocity $P(v_x)$ versus $v_x$ (black) and $P(v_y)$ versus $v_y$ (red) in the simulation (main panel) and experiment (inset). Bottom: contour maps of $P(v_x,v_y)$, with simulation in main panel and measurement in inset, for ants that are further than 3 cm from a boundary (left plot) or within 3 cm of a boundary (right plot)

In the upper left main panel of Fig. 3 we show the model densities *n*(*x*) versus *x* and *n*(*y*) versus *y*. The inset shows the experimental results for the same quantities. Note the enhanced value for the measured density *n*(*y*) near $y=L/2$, the deviation from square symmetry that we ascribed to the presence of the laboratory window. The model density respects square symmetry exactly.

The model density has the same qualitative behavior as the observed value: it is large at the boundary, then decreases over a length scale $\ell$ to a value that is constant throughout the arena interior. The analytic calculation (see Appendix B) of the 1D version of the model suggests that *n*(*x*) has an integrable divergence at the boundary and decreases exponentially. That is, at a small distance $d=L/2-x$ from the right boundary(or $d=-L/2+x$ from the left) we have $n(x)\sim d^{-2/3}$ for $d<<\ell$ while $n(x)-n(0)\approx \textrm{e}^{-d/\ell }$ for $d>>\ell$.

This behavior of *n*(*x*) is simply understood. Any ant that arrives at a boundary tends to remain nearby because, after it stops, it is improbable that a sequence of impulses in the same direction (away from the boundary) will carry it deep into the interior. More probably, it wanders a short distance from the boundary before returning and stopping again. This gives an enhancement of *n*(*x*) within distance $\ell$ of boundary. Members that are much further than $\ell$ have little chance of wandering far enough in one direction to encounter the boundary, so they behave as if it did not exist.

The velocity distributions $P(v_x)$ and $P(v_y)$ are plotted in the upper right of Fig. 3, with the model result in the main panel and experimental values in the inset. The overall distribution $P({\textbf {v}})$ exhibits square symmetry, so $P(v_x)=P(v_y)=P(-v_x)$. The function $P(v_x)$ is sharply peaked at $v_x=0$ and initially decreases with $|v_x|$. This peak is dominated by ants moving along $y=\pm L/2$ boundaries that frequently have the normal component of their velocity reset to zero. After reaching a local minimum, $P(v_x)$ recovers to exhibit shoulder peaks that are characteristic of the interior population. They are centered near $|v_x|\approx v_s$ with a half-width $v_\infty$. The shape of the simulated distribution resembles that of the experiment, at least all features are represented. From Appendix A, in which we allow model parameters to vary, we get an indication that the observed $P(v_x)$ function might be fully reproduced within this model framework.

A 2D representation of $P({\textbf {v}})$ is illustrated in the lower panels of Fig. 3 using contour plots for the model (main panels) and experiment (insets). On the left we show $P({\textbf {v}})$ for ants in the arena interior (more than 3 cm from the boundary). The distribution is isotropic, with a valley and ring-shaped plateau seen at finite speed (this is the 2D realization of the shoulders). The experimental result, while more ragged, has the same qualitative character. On the right we plot $P({\textbf {v}})$ for ants in the 3 cm wide boundary strip. Since they remain close to the boundary, they move rapidly along it, giving the characteristic four-lobed shape.

It should be emphasized that model parameters were obtained from the homogeneous, isotropic distribution of $\Delta {\textbf {v}}$ within the arena interior. The variation of $n({\textbf {x}})$ near the boundary, and features of $P({\textbf {v}})$, including any direction-dependence, were not assumed within the model but emerged as calculated properties of it. The basis for our claim that the model is in semi-quantitative agreement with the data is that the model length scale $\ell$ and speed scales $v_\infty$, $v_0$ are consistent with observed values.

The density $n({\textbf {x}})$, being strongly peaked at the arena boundaries, exhibits the signature of wall-following, a well-known property of ant motion [51]. An animal that exhibits active wall-following behavior should show a marked preference to remain near the wall and move differently when close to a wall than it does in open space. In Ref. [54] the motion of a cockroach was parameterized separately for phases when the animal was in the arena interior and when it was within antenna-length of the boundary. The harvester ants studied in Ref. [55] were observed to spend most of their time in the boundary region when confined to an arena similar to ours. Their corresponding model included parameters characterising diffusive motion in the arena interior and independent parameters associated with the probability of an ant leaving the boundary region. Note that the open-arena motion of Ref. [55] differs somewhat from our picture since it took velocity direction to change randomly while speed remained constant whereas, based on the measured distribution $p(\Delta {\textbf {v}})$ for weaver ants, we varied both direction and speed.

Our model ants cannot walk beyond an arena boundary so they stop when they reach the edge. But when they resume moving they are just as likely to turn away from the boundary as towards it. In fact, we slightly biased the model by forcing the ant at the boundary to take its first step into the arena interior. The model ants have no preference to remain close to the boundary, it is simply that the diffusive motion they undergo in the open arena does not effectively lead them away from its edge. As such, the fact that the simulated density $n({\textbf {r}})$ is peaked at the boundary results from passive wall-following behavior exhibited by model ants. But we can look further for evidence of active wall-following behavior in real weaver ants, i.e for a position-sensitive movement algorithm, by examining the distribution of resident times for individuals in the boundary strip.

We extracted the distribution $P(t_b)$ for the time $t_b$ an ant remains continuously in the 3 cm boundary strip after visiting the arena edge. Specifically, we logged all times $t=t_1$ when the ant displacement achieved a local maximum |*x*(*t*)| within 0.5 cm of the $|x|=L/2$ boundary and the time $t=t_2=t_1+t_b$ when it subsequently moved beyond the 3 cm boundary strip. Accordingly, $t=t_2$ is the earliest time $t>t_1$ for which $|x(t)<$12 cm. Note that an ant whose center of mass comes within 0.5 cm of the arena edge, half the length of its body, can reasonably be said to have contacted the boundary [61]. We consider only the *x* coordinate for this distribution in order to avoid having to make special provisions for arena-corner effects.

**Fig. 4:**
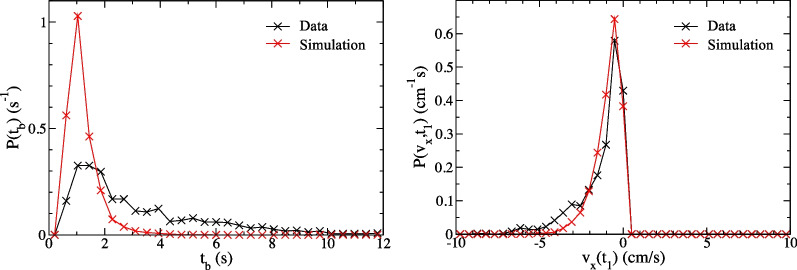
The normalized distribution $P(t_b)$ for the time $t_b$ needed for an ant to move from the boundary to the arena interior. That is $t_b=t_2-t_1$ where the ant contacted the arena boundary at $t=t_1$ and exited the 3 cm wide boundary strip at $t=t_2$ (further details are described in the text). In the left panel the experimental $P(t_b)$ is compared to the model result. In the right panel we show the distributions $P(v_x,t_1)$ of the *x* component of velocity $v_x(t_1)$ at time $t=t_1$ where positive values correspond to motion towards the boundary

The normalized distribution $P(t_b)$ is shown in the left panel of Fig. 4 along with corresponding model prediction. The latter was obtained from simulation data using the same procedure followed for the experimental data. The complementary distributions $P(v_x,t_1)$ of velocity perpendicular to the boundary at $t=t_1$ are included in the right panel. Note that, since $x(t_1)$ is a maximum, all ants have a velocity $v_x(t_1)$ that is negative, i.e. towards the interior, or zero. The model distribution $P(t_b)$ decays with time $t_b$ on the scale of approximately $\tau _D$. The experimental distribution also drops significantly over this time scale, confirming that the high density $n({\textbf {r}})$ at the boundary can largely be explained by the passive wall-following behavior present in the model. But there is a long tail in the measured distribution, giving a significantly higher probability $P(t_b)$ for large $t_b$ values in the experiment than in the model simulation.

The tail of the $P(t_b)$ distribution can be attributed to an additional tendency for active wall-following present among real weaver ants. As noted above, such an effect could be incorporated into the model using a position-dependent force term in Eq. 6. While our current interest is the population distribution shown in Fig. 3, we note that in performing fits of a parameterized force ${\textbf {F}}({\textbf {r}},{\textbf {v}})$ to data like that of Fig. 4, our model ants provide a control group that establishes the baseline distribution $P(t_b)$ in the absence of active wall-following. The force parameters obtained for different experimental groups, perhaps those with ants following a tactile wall instead of the arena boundary used here, could thus be more meaningfully compared.

The movement algorithm that the ants employ during the conditions of the experiment appears to be simplistic. The ants can be, at most, weakly influenced by long-range sensory input, such as their view of the panorama, measurement of light polarization or other sources. We saw that $P({\textbf {v}})$ and, to a lesser extent, $n({\textbf {x}})$ exhibited square symmetry. (The enhanced $n({\textbf {x}})$ for the arena edge nearest the window was a quantitative, not qualitative, symmetry violation.) This symmetry is an indication that ants were using local information to navigate, rather than looking beyond the arena for guidance. For, while the arena itself (an average over square tiles) was square-symmetric, the panorama of the laboratory was not.

There is also no indication that our ants were searching the arena in a systematic way, such as turning in widening circles. First, they do not favor one turning direction over another, as you would expect for those turning circles with a certain sense of rotation. More importantly, the correlation between consecutive impulses normal to velocity is negligible. If an ant was following a smooth trajectory then its velocity change during a given time step would be strongly correlated with that of the previous step. Instead, it appears that the ant is changing its velocity haphazardly.

The experiment subjected the weaver ant to unnatural conditions, and it is possible that the ant was unable to utilize its usual navigational tools within the confined arena. This might explain its apparently confused motion. The results above would then have little to say about the dispersion of weaver ants in nature. We are currently [62] using the same experimental configuration with a pair of ants, and studying whether the frequent interactions between the partners influence their individual motion. So, in this worst case, the model could still prove useful for studying communication among weaver ants.

On the other hand, if the observed motion is representative of weaver ant movement in natural situations then it raises questions about how motion described by the model above could be of any tactical advantage. In a homogeneous environment where it is difficult to keep track of one’s position, a systematic search pattern might be difficult to follow. An individual searching for a faint chemical trail on a barren landscape might behave like a Brownian particle, which tends to remain localized near a starting point but does not repeatedly follow the same path [63–65]. Such randomized motion would, eventually, leave no stone unturned.

## Conclusions

We considered the motion of an individual weaver ant moving in a square arena. Experimental data on the ant distribution over position and velocity were presented. We developed a model to explain the observed results. The distributions were non-trivial: the ant showed a strong tendency to remain near the arena boundaries and its distribution over speed was non-monotonic with several characteristic features.

The model assumed that the ant undergoes random velocity change according to a fixed probability distribution and that it has no preference whatsoever for position within the arena or direction of motion. With these minimal assumptions and without adjustable parameters, the model captures the data semi-quantitatively. The work provides insight into the movement algorithm used by ants when confined in an unfamiliar region.

## Figures and Tables

**Fig. 5:**
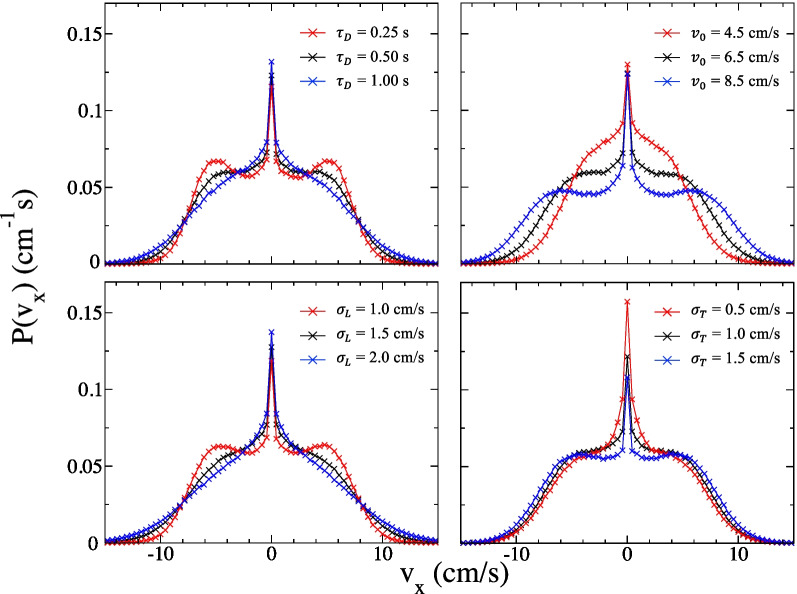
Comparing the simulated velocity distribution $P(v_x)$ by varying parameter values. In each panel only one parameter will be changed and the otherwise parameters are remain as in Fig. 4. Top left: the shoulder features are altered by varying $\tau _D = 0.25$ s (red), 0.5 s (black), 1.00 s (blue). Top right: the shoulders are displaced according to the driving force $v_0 = 4.5$ cm/s (red), 6.5 cm/s (black) and 8.5 cm/s (blue). Bottom left: changing $\sigma _L = 1$ cm/s (red), $\sigma _L = 1.5$ cm/s (black) and $\sigma _L = 2$ cm/s (blue) has a similar effect to varying $\tau _D$. Bottom right: Changing $\sigma _T = 0.5$ cm/s (red), 1.0 cm/s (black) and 1.5 cm/s (blue) has little effect on the shoulders but affects the central peak

**Fig. 6:**
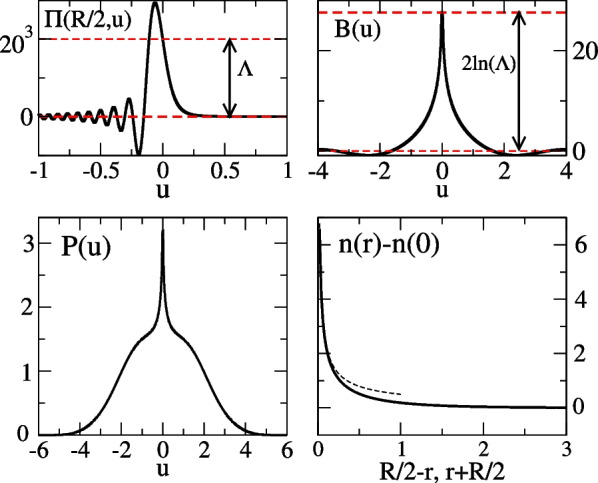
Upper left: the equilibrium distribution $\Pi (r,u)$ at the right boundary *R*/2. Its peak height scales with the cutoff $\Lambda$ and its width scales with $\Lambda ^{-1/3}$. Here we used $\Lambda =20^3$ for illustration. Upper right: the function *B*(*u*) that controls the contribution of arena boundaries to the velocity distribution. Its peak height and width scale as $2\ln \Lambda$ and $1/\ln \Lambda$, respectively. We used $\Lambda =10^6$ in this plot. Bottom left: the velocity distribution *P*(*u*), with the parameter $\alpha =1.5$. Bottom right: the density $n(r)-n(0)$ is plotted versus the distance *d* from the right boundary $d=R/2-r$ (or left boundary $d=r+R/2$) using $u_0=1.5$. The dashed line is the analytic approximation, for $d<<1$, in Eq. 39

## Data Availability

Experimental data and numerical codes available upon request from authors.

## Appendix A: variation of distribution with model parameters

To better understand the range of behavior possible for the model, we relax the requirement that parameters must be fixed to measured values. As described above, the density in the interior is constant by symmetry. It formally diverges at the boundary since the current of ants incident on the $x=L/2$ boundary during a given time step will all be placed at $x=L/2$ for the next time step: a finite number of members occupying a single point. The value of the basic distance scale $\ell$, over which *n*(*x*) varies, changes as we modify parameters: larger mean-square impulses $\sigma _L^2$ and a smaller drag force coefficient $1/\tau _D$ increase the region that a given ant explores, extending the range over which boundaries influence the distribution. But the qualitative behavior of the *n*(*x*) does not change dramatically.

The distinctive shape of $P(v_x)$ with round shoulder peaks coming from the interior and a sharp central peak from the boundaries, is sensitive to parameter values. In Fig. 5 we show the distribution for various parameter choices. In the upper panels we vary $\tau _D$ and $v_0$, which control the deterministic force, and in the lower panels vary $\sigma _L$ and $\sigma _T$, the size of the random impulses.

The shoulder peaks are centered on $\pm v_0$ and have a width $v_\infty$. Increasing the driving force $v_0$ pushes them outward without changing their height. Upon decreasing $v_0$, the shoulders can merge with the central peak, so the width of the latter increases significantly. If the drag coefficient $1/\tau _D$ increases then accumulated random impulses cannot achieve large speeds so $v_\infty$ is decreased. The result is the shoulder peaks become narrow, tall and more pronounced. On the other hand, members become more narrowly localized in space so $\ell$ decreases and the boundaries affect a smaller region. The height of the central peak slightly decreases as a result. If $1/\tau _D$ decreases and $v_\infty$ gets large then the shoulders become wider and shorter, merging into a smooth distribution.

With the deterministic force fixed we can vary the size of the root-mean-square random impulses $\sigma _L$ and $\sigma _T$. Variation of $\sigma _L$ has a similar effect to changing $\tau _D$, as is clear from the two plots on the left of Fig. 5. This is because the distribution results from a competition between the deterministic force, which scales as $1/\tau _D$, and the random impulses that scale as $\sigma _L^2$. So, increasing one of these is similar to decreasing the other. Recall, in Eq. 19, the anisotropy of the impulses produced only logarithmic corrections to the distribution in the interior. As a result, when we change $\sigma _T$ the shoulder features look the same, as seen in the lower right of Fig. 5. But the central peak is more sensitive to $\sigma _T$. After a model ant hits the boundary its body is aligned parallel with it (because the normal component of its velocity is reset to zero). If $\sigma _T$ is small, it cannot change direction effectively so it moves along the edge and remains in contact with the boundary. The height of the central peak, which indicates the weight of the boundary population, increases as a result.

## Appendix B: Equilibrium distribution for a 1D arena

Since the rate equation for the bounded 2D model is more difficult to solve, we consider a simpler 1D model in this section. The solution for the 1D equilibrium distribution reproduces the key properties of the density *n*(*x*) and velocity distribution $P(v_x)$ found above in the 2D simulations and experiment. The 1D model can be viewed as the limiting case of extremely anisotropic impulses for the 2D model. We set $\sigma _T=0$ so an ant initially oriented along the *x* axis remains so.

The arena is one-dimensional with length $R=L/\ell$ centered on the origin $x=0$, with parameters for speed $v_\infty$ and distance $\ell$ defined in Eq. 16. The position and velocity are *x* and *v* have dimensionless counterparts $r=x/\ell$ and $u=v/v_\infty$. The force *F* and its dimensionless version *f* are

23 $$\begin{aligned} F=-\frac{v}{\tau _D}+\frac{v_0\textrm{sign}(v)}{\tau _D},\;\;\;\;f=-u+u_0\textrm{sign}(u) \end{aligned}$$

and the rate equation that determines the equilibrium distribution $\Pi (r,u)$ is

24 $$\begin{aligned} 0=\frac{\partial }{\partial u}\bigg (-f\Pi +\frac{\partial }{\partial u}\Pi \bigg )-u\frac{\partial }{\partial r}\Pi . \end{aligned}$$

Following the protocol for the simulations described above, we know that the boundary distribution $\Pi (R/2,u)=\Pi (-R/2,-u)$ should be similar to $\delta (u)$ because all members that reach a boundary are restarted with zero velocity. However, at a boundary, the distribution is not an even function of *u*.

To solve Eq. 24 it is helpful first to find eigenfunctions $g_\lambda (u)$ of the velocity operator appearing in it, in the sense that

25 $$\begin{aligned} \bigg (-f g_\lambda (u)+g'_\lambda (u)\bigg )'=\lambda u g_\lambda (u),\;\;\textrm{with}\;g_\lambda (0)=1 \end{aligned}$$

for a given eigenvalue $\lambda$. (The primes denote derivatives with respect to *u*.) Having found such functions, we can write the general solution for the equilibrium distribution as a sum over $g_\lambda (u)$ functions weighted by coefficients $c_\lambda (r)$ that vary exponentially with position,

26 $$\begin{aligned} \Pi (r,u)=\sum _\lambda c_\lambda (r)g_\lambda (u)\;\;\textrm{where}\; c_\lambda (r)=c_\lambda (R/2)\textrm{e}^{\lambda [r-R/2]}. \end{aligned}$$

Also, Eq. 25 implies $g_{-\lambda }(u)=g_\lambda (-u)$ and the equilibrium distribution must satisfy reflection symmetry so $\Pi (r,u)=\Pi (-r,-u)$. This means we can restrict the sum to non-negative eigenvalues and write

27 $$\begin{aligned} \Pi (r,u)=\sum _{\lambda \ge 0} c_\lambda (R/2)\bigg (g_\lambda (u)\textrm{e}^{\lambda [r-R/2] }+g_\lambda (-u)\textrm{e}^{-\lambda [r+R/2]}\bigg ). \end{aligned}$$

The $\lambda =0$ term is position-independent: it gives the equilibrium distribution for an unbounded arena. The $\lambda >0$ terms vary with position: the first term on the right of Eq. 27 dominates near the right boundary but is negligible at the left boundary. The second term has the opposite property.

The eigenvalue equation, Eq. 25, for $\lambda =0$ requires that

28 $$\begin{aligned} -f(u)g_0(u)+g_0'(u)=-f(\infty )g_0(\infty )+g'_0(\infty )=0, \end{aligned}$$

where the last equality is required for a (normalizable) physical distribution. It is solved by

29 $$\begin{aligned} g_0(u)=\exp (\chi _0[u])\;\;\textrm{with}\;\;\chi _0(u)=\int _0^u du' f(u')=-\frac{u^2}{2}+|u|u_0. \end{aligned}$$

Clearly, $g_0(u)$ is the 1D analogue of the equilibrium distribution found above, in Eq. 19, for the 2D unbounded arena. According to Eq. 27, the distribution approaches $g_0(u)$ as one moves further from a boundary.

The eigenfunction $g_\lambda (u)$ for $\lambda \ne 0$ can be expressed exactly as a Hermite function, with an order that increases with $\lambda$, multiplied by a Gaussian factor. It oscillates increasingly rapidly as $\lambda$ increases, so a $g_\lambda (u)$ function is analogous to a Fourier component. Near the boundary, many $g_\lambda (u)$ contribute and their sum gives a velocity distribution that is sharply peaked near $u=0$.

To make it easier to sum over $g_\lambda (u)$ with $\lambda >0$ we approximate these functions. Consider Eq. 25 when $\lambda>>1$ and the terms expected to dominate it satisfy $g''_\lambda (u)-\lambda u g_\lambda (u)=0$. This is solved by an Airy function of the first kind: $g_\lambda (u)\propto \textrm{Ai}(\lambda ^{1/3}u)$. (An Airy function of the second kind also solves this equation but would give a physically incorrect distribution.) We substitute a trial form $g_\lambda (u)=\textrm{Ai}(\lambda ^{1/3}u)\exp (\chi _1[u])$, with $\chi _1(u)$ an unknown function, into Eq. 25 and obtain

30 $$\begin{aligned} 0=\lambda ^{1/3}\textrm{Ai}'(\lambda ^{1/3}u)\bigg (-f(u)+2\chi _1'(u)\bigg )+\textrm{Ai}(\lambda ^{1/3}u)\bigg (-f'(u)-f(u)\chi _1'(u)+\chi _1''(u)+[\chi _1'(u)]^2\bigg ). \end{aligned}$$

For sufficiently large $\lambda$ the first expression in parentheses, which is multiplied by $\lambda ^{1/3}$, must vanish. It does if $\chi _1(u)=\chi _0(u)/2$. The result

31 $$\begin{aligned} g_\lambda (u)=\exp (\chi _0[u]/2)\frac{\textrm{Ai}(\lambda ^{1/3}u)}{\textrm{Ai}(0)} \end{aligned}$$

agrees reasonably well with the true eigenfunction for $\lambda \approx 1$ and becomes exact in the limit of large $\lambda$. It is convenient because it is well behaved for any eigenvalue so we can treat $\lambda >0$ as a continuous parameter. Note that $\textrm{Ai}(0)=(3^{2/3}\Gamma [2/3])^{-1}\approx 0.36$ and the Airy function is positive and exponentially decaying when its argument is positive but oscillates in sign for negative argument.

We replace the sum over non-zero $\lambda$ with an integral using an interpolating function $c(\lambda )\equiv c_\lambda (R/2)$. A minimum value of $\lambda =1$ will be imposed on the continuous spectrum–the precise minimum value will not affect important results below. The distribution becomes:

32 $$\begin{aligned} \Pi (r,u)=c_0\textrm{e}^{\chi _0(u)}+ \frac{\textrm{e}^{\chi _0[u]/2}}{\textrm{Ai}(0)}\int _{1}^{\Lambda } d\lambda c(\lambda )\bigg ( \textrm{Ai}(u\lambda ^{1/3})\textrm{e}^{\lambda [r-R/2]}+\textrm{Ai}(-u\lambda ^{1/3})\textrm{e}^{-\lambda [r+R/2]}\bigg ). \end{aligned}$$

The cutoff of the $\lambda$ integral can be chosen according to the desired position resolution $\Delta r$ and velocity resolution $\Delta u$ of the distribution. The terms with $\lambda \gg 1/\Delta r$ are zero at any measurable distance from the boundary, so they can be ignored. Also, since $\textrm{Ai}(\pm \infty )=0$, terms with $\lambda>>1/\Delta u$ contribute nothing to the distribution. For our, qualitative, purpose we set $\Lambda =\infty$ whenever the integral converges. Also, if the $\lambda =0$ term does not dominate the sum then we can extend the lower integration limit to $\lambda =0$ (it does not matter if we use Eq. 31 to poorly approximate $g_0(u)$ in this case).

The $c(\lambda )$ coefficients should be determined by matching the $r=R/2$ distribution

33 $$\begin{aligned} \Pi (R/2,u)=\frac{\textrm{e}^{\chi _0(u)/2}}{\textrm{Ai}(0)} \int _{0}^{\Lambda } d\lambda c(\lambda )\textrm{Ai}(u\lambda ^{1/3}),\;\;\;\Pi (R/2,0)=\int _0^\Lambda d\lambda c(\lambda ) \end{aligned}$$

to a known boundary value. Current conservation at the boundary determines the rate that members are replaced at $r=R/2$ with zero velocity. But as a rough approximation to this procedure, and in analogy with the corresponding situation for a Fourier transform, we assume that $u=0$ members at $r=R/2$ are uniformly distributed among all $\lambda$ components. This means $c(\lambda )=c_1\approx c_0$ with $c_1$ a constant.

The border distribution is now

34 $$\begin{aligned} \Pi (R/2,u)\approx \frac{3c_1}{\textrm{Ai}(0)u^3}\bigg (\textrm{Ai}(0)-\textrm{Ai}(\Lambda ^{1/3}u)+u\Lambda ^{1/3}\textrm{Ai}'(u\Lambda ^{1/3})\bigg ) \end{aligned}$$

where we used the fact that the integral is strongly peaked about $u=0$ to set $\chi _0(u)\approx \chi _0(0)=0$ and then used the defining differential equation of the Airy function to do the $\lambda$ integral. At $u=0$ the function has a value $\Pi (R/2,0)\approx \Lambda$ and is peaked at the slightly negative velocity of $u=u_{\max }\approx -1.47\Lambda ^{-1/3}$ with $\Pi (R/2,u_{\max })\approx 1.46\Lambda$. For positive velocities it drops to zero on a scale of $u_{\max }$ while for negative velocities it oscillates on this same scale, rapidly averaging to zero. In Fig. 6 we plot it using a modest value $\Lambda ^{1/3}=20$ so the qualitative behavior can be clearly seen.

The overall velocity distribution is similarly obtained

35 $$\begin{aligned} P(u)=\int _{-R/2}^{R/2} dr \Pi (r,u)=c_0R\textrm{e}^{\chi _0(u)}+c_1\textrm{e}^{\chi _0(u)/2}B(u) \end{aligned}$$

where

36 $$\begin{aligned} B(u)=\frac{3}{\textrm{Ai}(0)}\int _{1}^{\Lambda ^{1/3}} \frac{dz}{z} \bigg ( \textrm{Ai}(zu)+\textrm{Ai}(-zu)\bigg ). \end{aligned}$$

The function *B*(*u*) comes from the population near the boundaries and determines the velocity distribution at small *u*. It has a $u=0$ peak of height $B(0)=2\ln \Lambda$ and width (FWHM) of approximately $4/\ln \Lambda$. While *B*(*u*) depends on $\Lambda$, this dependence is weak. In Fig. 6 we plot *B*(*u*) and *P*(*u*) with $\Lambda =10^6$ and $u_0=1.5$, $R=4$ and $c_0=c_1$. (The vertical axis for plots of *P*(*u*) and *n*(*r*) is arbitrary in this figure: the amplitude of these distributions should be fixed by normalization.) The cusp in *P*(*u*) at $u=0$ comes from that of the *B*(*u*) function while the shoulders, coming from $g_0(u)$, correspond to $u=\pm u_0$.

To obtain the density

37 $$\begin{aligned} n(r)=\int _{-\infty }^{\infty } du \Pi (r,u)\;\;\textrm{with}\;n(0)=c_0\int _{-\infty }^{\infty } du \textrm{e}^{\chi _o(u)} \end{aligned}$$

we integrate Eq. 32 numerically. The $\lambda$ integral converges so we can take $\Lambda \rightarrow \infty$. The result for $n(r)-n(0)$ is plotted in Fig. 6 versus the distance from either boundary.

Insight can be gained by considering the case of no driving force, i.e. setting $u_0=0$, so that analytic results for *n*(*r*) are possible. Integrating Eq. 32 over *u*, using the large $\lambda$ approximation of $2\sqrt{\pi }\textrm{e}^{2\lambda ^2/3}\textrm{Ai}(\lambda ^{4/3})\approx \lambda ^{-1/3}$, and taking $\Lambda \rightarrow \infty$ we find

38 $$\begin{aligned} n(r)-n(0)\approx c_1\int _1^\infty d\lambda \lambda ^{-1/3} \bigg (\textrm{e}^{\lambda (r-R/2)}+\textrm{e}^{-\lambda (r+R/2)}\bigg )\;\;\;\textrm{for}\;u_0=0. \end{aligned}$$

Writing the positive distance from the left boundary as $d=R/2+r$ or from the right boundary as $d=R/2-r$, we have

39 $$\begin{aligned} n(r)-n(0)\approx \frac{c_1\Gamma (2/3)}{d^{2/3}}\;\; (\textrm{for}\;d<<1)\;\;,\; n(r)-n(0)\approx \frac{c_1\textrm{e}^{-d}}{d}\;\;(\textrm{for}\;d>>1). \end{aligned}$$

If we had extended the lower limit of the integral to $\lambda =0$ then the density, for all distances *d*, would be given by the $d<<1$ expression above (so the lower cutoff in the $\lambda$ integral affects the long-distance decay of the boundary population). In Fig. 6 we compare the $d^{-2/3}$ divergence with the numerical calculation of *n*(*r*). To within a constant multiplicative factor, the $u_0=0$ approximation of the density is similar as the result for finite $u_0$.

The results above are consistent with what we saw in the experiment and the model simulations. The density *n*(*x*) is large at the boundary because so many slow-moving ants remain near it. The density decreases with distance *d* and ants further than $\ell$ from the boundary act as though they are in an infinite arena. The sharp $v=0$ peak in the velocity distribution *P*(*v*) is due to ants at the boundary that are often abruptly stopped. The shoulder features in *P*(*v*) are properties of the distribution in the arena interior: they are local maxima in the distribution centered on $v=v_0$ with a width of $v_\infty$.
