# Correction: Modeling the movement of *Oecophylla smaragdina* on short-length scales in an unfamiliar environment

**DOI:** 10.1186/s40462-024-00456-y

**Published:** 2024-02-28

**Authors:** L. Charoonratana, T. Thiwatwaranikul, P. Paisanpan, S. Suksombat, M. F. Smith

**Affiliations:** 1 School of Physics, Suranaree University of Technology, 30000 Nakhon Ratchasima, Thailand; 2 NANOTEC-SUT Center of Excellence on Advanced Functional Nanomaterials, Suranaree University of Technology, 30000 Nakhon Ratchasima, Thailand; 3 School of Sport Science, Suranaree University of Technology, 30000 Nakhon Ratchasima, Thailand

**Correction to:** ***Movement Ecology*** **(2023) 11:64**

10.1186/s40462-023-00426-w

The original publication of this article contained a spelling mistake in the title and in the Abstract, Introduction and Experimental results sections. The species of ant was written incorrectly as *Oecophylla smargandina*, instead of *Oecophylla smaragdina*.

The original article has been updated.

